# Hemophagocytic Syndrome-Associated Variant of Methotrexate-Associated Intravascular Large B-Cell Lymphoma in a Rheumatoid Arthritis Patient

**DOI:** 10.1155/2019/8947616

**Published:** 2019-09-11

**Authors:** Yukiko Komeno, Minako Akiyama, Yasumi Okochi, Hitoshi Tokuda, Keiko Abe, Kuniko Iihara, Tomiko Ryu

**Affiliations:** ^1^Department of Hematology, Japan Community Healthcare Organization (JCHO) Tokyo Yamate Medical Center, Hyakunin-cho, Shinjuku, Tokyo 169-0073, Japan; ^2^Department of Nephrology, JCHO Tokyo Yamate Medical Center, Hyakunin-cho, Shinjuku, Tokyo 169-0073, Japan; ^3^Department of Respiratory Medicine, JCHO Tokyo Yamate Medical Center, Hyakunin-cho, Shinjuku, Tokyo 169-0073, Japan; ^4^Department of Pathology, JCHO Tokyo Yamate Medical Center, Hyakunin-cho, Shinjuku, Tokyo 169-0073, Japan

## Abstract

A 59-year-old man was treated for rheumatoid arthritis (RA) for 12 years with methotrexate (MTX) and prednisolone. After MTX-associated interstitial pneumonia developed, he was treated with cyclophosphamide and prednisolone for 7 months. Arthritis worsened, and tacrolimus was added to the treatment regimen. One month later, he had fever, loss of appetite, and dyspnea on exertion. Blood tests showed pancytopenia with large, atypical lymphocytes. Computed tomography showed mild splenomegaly. Bone marrow examination demonstrated CD20-positive, EBER-positive atypical lymphocytes, and hemophagocytosis. Random skin biopsy led to the diagnosis of intravascular large B-cell lymphoma (IVLBCL). The final diagnosis was a hemophagocytic syndrome-associated variant of IVLBCL. Complete remission was achieved after seven courses of R-CHOP. However, within a month, he complained of dizziness. Magnetic resonance imaging revealed focal infarctions in the cerebellum and around the left lateral ventricle. Central nervous system relapse was suspected. Although salvage chemotherapy (CHASER), whole brain irradiation, and intrathecal injection of cytarabine and prednisolone were temporarily effective, he died. Autopsy revealed infiltration of lymphoma cells in the brain and adrenal glands. To the best of our knowledge, this is the sixth case of IVLBCL and the first case of the hemophagocytic syndrome-associated variant of IVLBCL in RA patients in the literature.

## 1. Introduction

According to WHO classification, intravascular large B-cell lymphoma (IVLBCL) is a rare type of extranodal large B-cell lymphoma characterized by the selective growth of lymphoma cells within the lumina of vessels, in particular capillaries, with the exception of larger arteries and veins [1, 2]. It comprises three major subtypes: a classic variant, a hemophagocytic syndrome-associated variant, and a cutaneous variant. The former variants were originally known as Western and Asian variants, respectively, based on geographical distributions. Murase et al. proposed diagnostic criteria of the hemophagocytic syndrome-associated (Asian) variant [3]. IVLBCL disseminates to almost all organs, while lymphadenopathy is absent in most cases. The prognosis of IVLBCL is very poor, partly because disease presentation can be varied and nonspecific, which makes early diagnosis difficult. CNS relapse occurs in up to 25% of IVLBCL cases, and its outcome is dismal. Unlike the other two variants, the cutaneous variant is restricted to the skin and is associated with a better prognosis.

Lymphoid proliferations or lymphomas arising in patients treated with immunosuppressive drugs for autoimmune diseases, excluding transplantation recipients, are classified as “other iatrogenic immunodeficiency-associated lymphoproliferative disorders (OIIA-LPD)” according to WHO classification [2]. Rheumatoid arthritis (RA) is the most representative underlying disorder, typically treated with methotrexate (MTX). The most dominant histopathology of MTX-associated LPD is diffuse large B-cell lymphoma (DLBCL) and Hodgkin lymphoma [2]. As for IVLBCL arising in RA patients, only five cases have been reported in the literature [4–8].

Here, we present the first case of the hemophagocytic syndrome-associated variant of IVLBCL that developed during the follow-up of rheumatoid arthritis treated with MTX.

## 2. Case Presentation

A 43-year-old Japanese man was diagnosed with RA in 1995. In 1998, treatment with methotrexate (MTX) and prednisolone (PSL) was started. In November 2010, he was diagnosed with MTX-induced interstitial pneumonia, and MTX was discontinued. He was treated with pulse steroid therapy, followed by a tapering dose of prednisolone. In January 2011, cyclophosphamide (CPA) was added to treat dyspnea due to interstitial pneumonia. In July 2011, tacrolimus was added for the treatment of arthralgia. In the following months, hemoglobin and platelet levels gradually decreased, and lactate dehydrogenase (LDH) levels increased. Tacrolimus side effects were suspected, and tacrolimus was discontinued. Two weeks later (August 2011, at the age of 59), he visited the hospital for fever, appetite loss, dyspnea on exertion, and skin rash. Blood testing revealed anemia, thrombocytopenia, and elevated levels of LDH and CRP. He was hospitalized for further examination.

**Figure 1:**
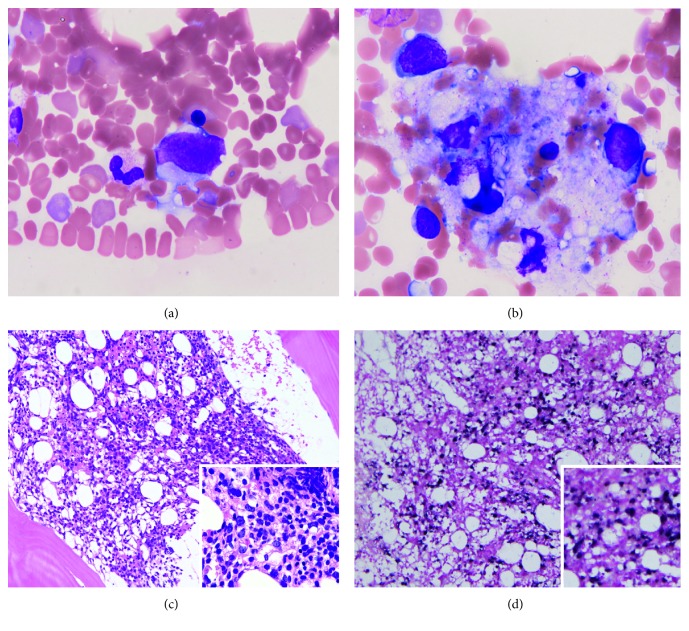
Bone marrow aspiration/biopsy findings. (a, b) Bone marrow aspirate. Wright-Giemsa staining. (a) A large, atypical lymphocyte (×1,000). (b) Hemophagocytosis (×1,000). (c, d) Bone marrow biopsy showing hypercellular marrow. (c) Hematoxylin and eosin staining. Low-magnification image (×40). Inset: high-magnification image (×200). (d) Epstein–Barr virus-encoded RNA (EBER) in situ hybridization. Large atypical lymphocytes are positive. Low-magnification image (×40). Inset: high-magnification image (×200).

**Figure 2:**
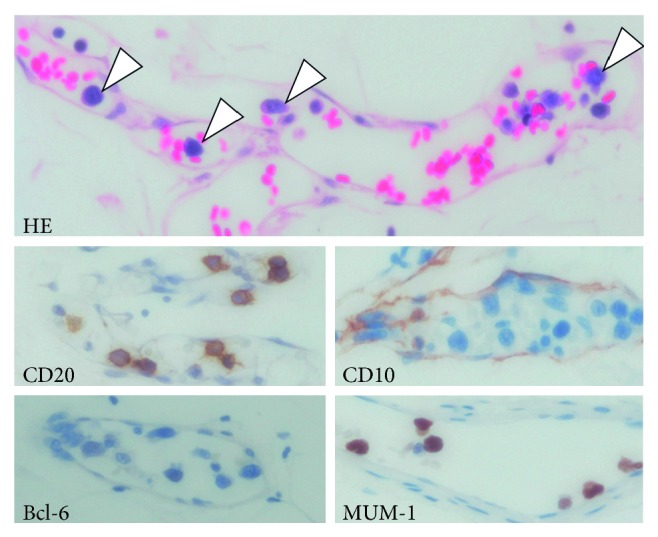
Histopathology of the angiolipoma. Large atypical lymphocytes in the lumina of the capillaries. Lymphoma cells are indicated by arrowheads. They are CD20(+), CD10(−), Bcl-6(−), and MUM-1(+). Magnification, ×400. HE, hematoxylin and eosin stain.

On admission, he was administered PSL 10 mg/day daily and CPA 50 mg/day every other day. Skin eruptions were observed on his back and lateral regions. Blood testing revealed pancytopenia with large atypical lymphocytes and elevated C-reactive protein levels (Table 1). Soluble interleukin-2 receptor (sIL-2R) was elevated to 12,600 U/mL. Blood culture was negative. Computed tomography (CT) showed inactive interstitial shadows in the subpleural region and mild splenomegaly but no lymphadenopathy. Bone marrow aspiration demonstrated atypical lymphocytes (3.0% of all nucleated cells) (Figure 1(a)) and hemophagocytosis by macrophages, suggesting lymphoma-associated hemophagocytic syndrome (Figure 1(b)). Bone marrow biopsy revealed hypercellular marrow with diffuse infiltration of large atypical lymphocytes, which were CD20(+), CD79a(+), Bcl-2(−), CD10(−), Epstein–Barr virus-encoded RNA (EBER) in situ hybridization (+), and high Ki-67 index level (100%) (Figures 1(c) and 1(d)). One of the four samples of random skin biopsy was angiolipoma containing relatively large atypical lymphocytes within the lumina of vessels (CD20(+), CD79a(+), CD10(−), Bcl-6(−), MUM-1(+), and Ki-67(+)) (Figure 2). These findings fulfilled the diagnostic criteria of the hemophagocytic-syndrome associated (or Asian) variant of IVLBCL (International Prognostic Index (IPI) high intermediate) [3].

Pulse steroid therapy (methylprednisolone 500 mg/day for 3 days) was started on day 2 after admission, which relieved dyspnea. After the diagnosis of IVLBCL, one course of CHOP (cyclophosphamide, doxorubicin, vincristine, and prednisone) and six courses of R-CHOP (rituximab plus CHOP) were administered. Splenomegaly was resolved, and blood cell counts returned to the normal levels. Normal hematopoiesis was confirmed by bone marrow biopsy. However, at the final (sixth) R-CHOP therapy (January 2012) as an outpatient, he complained of staggering gait, which started one month earlier. He was afebrile. Magnetic resonance imaging (MRI) performed in February 2012 revealed a fresh infarction in the white matter around the left lateral ventricle, which was possibly caused by IVLBCL. He was hospitalized. Laboratory findings were as follows: WBC 2,040/*μ*L, neutrophil 1,300/*μ*L, hemoglobin 14.0 g/dL, platelet 10.0 × 10^4^/*μ*L, LDH 241 IU/L, ferritin 141.4 ng/mL, and sIL-2R 319 U/mL. The finger-to-nose test was positive on the right side. Cytology of cerebrospinal fluid (CSF) was class II. Bone marrow aspiration/biopsy revealed no evidence of lymphoma or hemophagocytosis. As he had a past history of MTX-induced interstitial pneumonia, cyclophosphamide, cytosine arabinoside, dexamethasone, etoposide, and rituximab (CHASER) instead of high-dose MTX was administered as salvage therapy.

**Figure 3:**
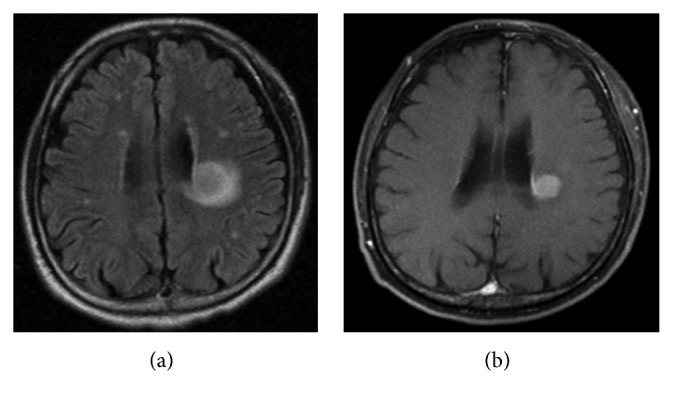
Magnetic resonance imaging (MRI) for CNS relapse. (a) Fluid-attenuated inversion recovery image showing a 2.3-cm mass in the infarction lesion in the white matter near the left lateral ventricle. (b) Contrast-enhanced MRI showing an evenly enhanced mass.

In March 2012, he was hospitalized for the second course of chemotherapy. On admission, MRI revealed a new 2.3-cm mass in the infarction lesion in the white matter around the left lateral ventricle (Figures 3(a) and 3(b)). It had low intensity in the T1-weighted image, higher intensity than cerebral parenchyma in the T2-weighted image (T2WI) and fluid-attenuated inversion recovery (FLAIR) (Figure 3(a)), and high intensity in the diffusion weighted image, and its apparent diffusion coefficient value was low. It was evenly enhanced in contrast-enhanced MRI (Figure 3(b)). These results suggested infiltration of lymphoma. Cytology of CSF was class II. The second course of CHASER was administered, followed by whole brain irradiation, and MRI revealed shrinkage of the mass.

In May 2012, he had staggering gait, headache, and dysesthesia in the left leg. MRI showed that the mass was smaller than it was before treatment, but it still had high intensity in T2WI/FLAIR and diffusion enhanced images, while no enhancement was noted in contrast-enhanced images. Cytology of CSF was class V. CNS relapse of IVLBCL was suggested. High-dose cytarabine and prednisolone therapy was administered for 3 days, and repeated intrathecal injections of cytarabine and prednisolone were administered. On MRI, the low-density area remained the same size and showed no enhancement on contrast-enhanced images. Continuous injection of oxycodone was administered for headache. In June 2012, dizziness, nausea, paresis of the right oculomotor nerve, and left leg paralysis manifested. Abdominal CT showed swelling of the bilateral adrenal glands, suggestive of infiltration of lymphoma cells, but neither lymphadenopathy nor hepatosplenomegaly was detected. Within the next two weeks, he had high fever and developed shock. Chest radiography revealed bilateral diffuse pneumonia. Despite antibiotic therapy and intrathecal injections of cytarabine and prednisolone, he died in August 2012, one year after diagnosis.

**Figure 4:**
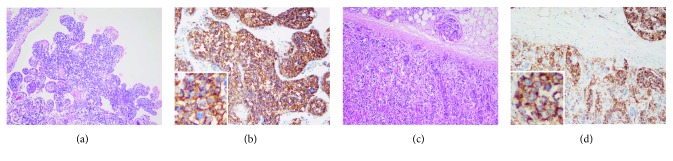
Autopsy findings. (a, b) Choroid plexus. (a) Hematoxylin and eosin staining. Magnification, ×100. (b) CD20 staining. Magnification, ×200. (c, d) Adrenal gland. (c) Hematoxylin and eosin staining. Magnification, ×100. (d) CD20 staining. Magnification, ×200.

Autopsy was performed with written consent from the patient's family. Multiple focal pneumonias and diffuse alveolar damage were noted in both lungs. Massive purulent sputum was found in the right main bronchus. Lymphoma infiltration was prominent in the brain and adrenal glands. Spotty red changes were observed around the left lateral ventricle, but no infarction, hemorrhage, or tumor formation was noted in the brain. Lymphoma cells were found in capillaries in the choroid plexus (Figures 4(a) and 4(b)) and pituitary gland. Bilateral adrenal glands were enlarged. Lymphoma cells were observed in the capillaries of the bilateral adrenal glands and fatty tissue around them (Figures 4(c) and 4(d)), ectopic adrenal cortex tissue around the spleen, and transverse colon. The lymphoma cells were CD20(+), CD79a(+), CD10(−), Bcl-6(−), MUM-1(+), CD5(−), CD3(−), and CD56(−). These findings were compatible with IVLBCL. No lymphoma infiltration was observed in the thyroid gland, liver, spleen, heart, skin, or bone marrow. No lymphadenopathy was detected. These results suggested that the cause of death was diffuse infiltration of IVLBCL in the brain and respiratory failure due to bilateral pan-lobular pneumonia.

## 3. Discussion

RA is associated with an increased risk of lymphoma [9]. Although its pathogenesis remains unclear, the increase in this risk appears to be related to the high inflammatory activity observed in RA, immunosuppressive agents, and Epstein–Barr virus infection. DLBCL is the most common subtype of lymphoma in RA [9]. Here, we reported a case of the hemophagocytic syndrome-associated variant of IVLBCL following immunosuppressive treatment for RA. To our knowledge, this is the sixth case of IVLBCL in RA reported in the literature [4–8]. Four cases, including ours, were from Japan [4–6]. However, of the six cases, ours is the only one involving the hemophagocytic syndrome-associated variant. This variant is characterized by bone marrow involvement, fever, hepatosplenomegaly, thrombocytopenia, and hemophagocytic syndrome [1–3].

Prior treatments for IVLBCL in RA patients have varied. Since most RA patients with OIIA-LPD have a history of treatment with MTX, the term “MTX-associated LPD” is commonly used. However, in most cases, several immunosuppressive agents were concomitantly used (such as PSL, etanercept, tumor necrosis factor inhibitor, and adalimumab) [4, 5, 7]. Our patient had MTX-associated interstitial pneumonia, and the prolonged use of PSL and CPA, and the use of tacrolimus for a month, was necessary after MTX cessation. In fact, MTX has not been proven to have a direct causative effect for lymphoma in RA patients [9]. Thus, the significance of the diagnosis of “MTX-associated LPD” needs further validation. In contrast, Willemze et al. reported on an RA patient who had been treated with only PSL 10 mg for 9 years and later developed IVLBCL [8]. The latency of IVLBCL in RA patients has ranged from 2 months [6] to 13 years (our case) after starting MTX treatment [4–6].

Approximately 40% of MTX-associated LPD cases show at least partial regression after drug withdrawal [2]. However, all the previously reported IVLBCL (classic variant) cases in RA patients were treated with chemotherapy. The outcomes were favorable, although the observation periods were relatively short. The clinical course of our case lasted for only one year, and massive infiltration of lymphoma cells into the CNS was noted during the autopsy; this is consistent with the fact that the hemophagocytic syndrome-associated variant shows a poor prognosis [3].

As IVLBCL cells proliferate in blood vessels, R-CHOP is the standard therapy [1]. However, CNS invasion/relapse is one reason for the poor prognosis of IVLBCL [1, 2]. CNS invasion/relapse was reported in three previous RA-associated IVLBCL cases as well as in ours. Kikuchi et al. administered 8 courses of R-CHOP and 4 rounds of intrathecal (IT) injections (PSL, MTX, and cytarabine) [5]. Hagihara et al. administered six courses of R-THPCOP, three rounds of IT injections of MTX plus dexamethasone, and one course of high-dose MTX [4]. Kida et al. administered R-Hyper-CVAD/R-MA (rituximab plus cyclophosphamide, vincristine, doxorubicin, and dexamethasone, alternating with rituximab plus high-dose MTX and cytarabine) [6]. Our patient, who was initially treated with R-CHOP, experienced CNS relapse during the last course of R-CHOP. As our patient had a past history of MTX-associated interstitial pneumonia, high-dose MTX was not administered. After relapse, he received two courses of CHASER, whole brain irradiation, and repeated IT injections of cytarabine and PSL. The best way of CNS prophylaxis of IVLBCL is not clear [1]. According to the ESMO guideline on the management of “ultra-high-risk” lymphoma patients, IPI parameters (age > 60 years, high LDH levels, poor performance status, advanced disease stage, and more than one extranodal site) are risk factors for early CNS relapse following first-line treatment of DLBCL including IVLBCL [10]. In addition, the involvement of the testes, kidneys, adrenals, breast, bone marrow, and bone increases the risk of CNS disease [10]. Thus, our patient, with a high LDH level and bone marrow invasion at diagnosis, should have benefitted from CNS prophylaxis. Intrathecal injections of MTX, cytarabine, and prednisolone have been the traditional method of CNS prophylaxis. However, significance of IT chemotherapy is controversial, because it does not reach measurable concentrations of brain parenchyma [10]. Instead, intravenous prophylaxis, such as systemic high-dose MTX, is an alternative strategy of CNS prophylaxis [1, 10].

In our case, random skin biopsy resulted in the diagnosis of IVLBCL. Among the four biopsies, the only sample showing positive results contained angiolipoma. There are two previous reports of IVLBCL diagnosed by angiolipoma biopsies [11, 12]. Biopsies of the lung and kidney can also be used for the diagnosis of IVLBCL [3]. Moreover, we previously reported a case of IVLBCL diagnosed by colon biopsy [13].

In conclusion, here, we reported a rare case of the hemophagocytic syndrome-associated variant of IVLBCL that developed during RA treatment. IVLBCL should be considered as a differential diagnosis of pancytopenia during RA treatment or immunosuppressive therapy.

## Figures and Tables

**Table 1:** Summary of the patient's initial laboratory data.

White blood cells	3750/*μ*L
Monocyte	15.5%
Lymphocyte	10.0%
Basophil	0.0%
Eosinophil	0.0%
Segmented	51.0%
Band	20.0%
Metamyelocyte	0.5%
Myelocyte	0.5%
Atypical lymphocyte	2.5%
Red blood cells	299 × 10^4^/*μ*L
Hemoglobin	9.4 g/dL
MCV	90.6 fL
Reticulocytes	6.94 × 10^4^/*μ*L
Platelets	5.0 × 10^4^/*μ*L
TP	5.1 g/dL
Albumin	2.6 g/dL
LDH	1336 IU/L
AST	83 IU/L
ALT	23 IU/L
ALP	385 IU/L
*γ*-GTP	80 IU/L
TB	1.3 mg/dL
DB	0.3 mg/dL
BUN	22 mg/dL
Creatinine	0.8 mg/dL
CRP	8.2 mg/dL
Fe	101 *μ*g/dL
Ferritin	5854.7 ng/mL
sIL-2R	12600 U/mL
PT	84%
PT-INR	1.09
aPTT	33.2 sec
D-dimer	2.8 *μ*g/mL

Segmented, segmented neutrophil. Band, band neutrophil. MCV, mean corpuscular volume. TP, total protein. LDH, lactate dehydrogenase. AST, aspartate transaminase. ALT, alanine aminotransferase. ALP, alkaline phosphatase. *γ*-GTP, *γ*-glutamyltransferase. TB, total bilirubin. DB, direct bilirubin. BUN, blood urea nitrogen. CRP, C-reactive protein. Fe, iron. sIL-2R, soluble interleukin-2 receptor. PT, prothrombin time. PT-INR, the international normalized ratio of prothrombin time. aPTT, activated partial thromboplastin time.
